# Supplementary material for: Cloud BioLinux: pre-configured and on-demand bioinformatics computing for the genomics community
Source: BMC Bioinformatics. 2012 Mar 19;13:42. doi: 10.1186/1471-2105-13-42 (PMC3372431; doi:10.1186/1471-2105-13-42)
Supplement: Additional file 1 — Supplementary 1 Cloud BioLinux software documentation in the form of a mini, self-contained website. Users need to download and uncompress the .zip file, and open through a web browser the "index.html" file available on the main directory. (ZIP 1823 kb). [file 1471-2105-13-42-S1.ZIP › Cloud-BioLinux-Package-Documentation/docs/blast+.html]

Bio-Linux Software Documentation Pages

Back to search form

## blast+

|  |  |
| --- | --- |
| Name | blast+ |
| Description | blast+ is a new version of the blast package from the NCBI.  The blast+ package consists of a number of stand-alone programs, for searching sequence databases, comparing two sequences, creating blast databases and masking low complexity sequence.  Benefits of the new package include:  - You can save search strategies and run them again later. - Flexible output of blast results. For example, you can get tab delimited output AND choose which columns of information are included. - blastx efficiency has apparently been increased. - blasting 2 sequences against each other is now easier  The main programs in the package for database searching are:  - blastn - blastp - blastx - psiblast - rpsblast+ - rpstblastn - tblastn - tblastx  Programs for creating and working with database include:  - makeblastdb - makembindex - blastdbcmd  Sequence masking utilities included in this package are:  - dustmasker - segmasker - windowmasker  Help on the options available for each program can be found by typing the program name followed by `-help` |
| Homepage | http://www.ncbi.nlm.nih.gov/bookshelf/br.fcgi?book=helpblast∂=CmdLineAppsManual |
| Remote Documentation | http://www.ncbi.nlm.nih.gov/bookshelf/br.fcgi?book=helpblast∂=CmdLineAppsManual   http://www.biomedcentral.com/1471-2105/10/4   Summary of command line options |

User manual for blast+ programs
